# *Symbiodinium* biogeography tracks environmental patterns rather than host genetics in a key Caribbean reef-builder, *Orbicella annularis*

**DOI:** 10.1098/rspb.2016.1938

**Published:** 2016-11-16

**Authors:** Emma V. Kennedy, Linda Tonk, Nicola L. Foster, Iliana Chollett, Juan-Carlos Ortiz, Sophie Dove, Ove Hoegh-Guldberg, Peter J. Mumby, Jamie R. Stevens

**Affiliations:** 1 College of Life and Environmental Sciences, University of Exeter, Stocker Road, Exeter EX4 4QD, UK; 2 Australian Rivers Institute, Griffith University, Nathan, 4111 Queensland, Australia; 3 Coral Reef Ecosystems Lab, School of Biological Sciences, University of Queensland, St Lucia, 4072 Queensland, Australia; 4 School of Marine Science and Engineering, Plymouth University, Drake Circus, Plymouth PL4 8AA, UK; 5 Smithsonian Marine Station, Smithsonian Institution, Fort Pierce, FL 34949, USA; 6 Marine Spatial Ecology Lab, School of Biological Sciences, University of Queensland, St Lucia, 4072 Queensland, Australia

**Keywords:** symbiont diversity, Zooxanthellae, environmental drivers, coral bleaching, denaturing gel gradient electrophoresis, Internal Transcribed Spacer 2

## Abstract

The physiological performance of a reef-building coral is a combined outcome of both the coral host and its algal endosymbionts, *Symbiodinium*. While *Orbicella annularis*—a dominant reef-building coral in the Wider Caribbean—is known to be a flexible host in terms of the diversity of *Symbiodinium* types it can associate with, it is uncertain how this diversity varies across the Caribbean, and whether spatial variability in the symbiont community is related to either *O. annularis* genotype or environment. Here, we target the *Symbiodinium*-ITS2 gene to characterize and map dominant *Symbiodinium* hosted by *O. annularis* at an unprecedented spatial scale. We reveal northwest–southeast partitioning across the Caribbean, both in terms of the dominant symbiont taxa hosted and in assemblage diversity. Multivariate regression analyses incorporating a suite of environmental and genetic factors reveal that observed spatial patterns are predominantly explained by chronic thermal stress (summer temperatures) and are unrelated to host genotype. Furthermore, we were able to associate the presence of specific *Symbiodinium* types with local environmental drivers (for example, *Symbiodinium* C7 with areas experiencing cooler summers, B1j with nutrient loading and B17 with turbidity), associations that have not previously been described.

## 1. Background

*Symbiodinium*, dinoflagellate endosymbionts present in many marine invertebrates, play key roles on tropical coral reefs. Their symbioses with reef-building corals underpin a diverse and productive ecosystem, enabling the establishment of reefs in oligotrophic tropical waters. *Symbiodinium* translocate organic compounds, generated by photosynthesis, to their coral hosts and can account for 50–70% of reef primary production [1]. Importantly, they also promote calcium carbonate skeletal deposition in their coral hosts, helping to build three-dimensional structure, a process vital to maintaining coral reef ecosystem function [2].

Despite their important role, the diversity and distribution of *Symbiodinium* taxa are still in the process of being described for many host organisms. Molecular techniques reveal diversity in excess of 400 taxa, nested within nine genetically divergent groups (clades A–I) in what was once thought to be a single pandemic species. The ecological consequences of this diversity are not fully understood [3], but evidence suggests that association with certain *Symbiodinium* species may affect aspects of coral colony physiology, principally calcification rate and bleaching thresholds [4]. With rising sea temperatures threatening the future of reefs globally [5], recognition of the variation in the coral–endosymbiont association is vital for understanding the capacity of corals to survive and maintain healthy reef growth in a rapidly changing climate [6].

Scleractinian corals predominantly host *Symbiodinium* clade C, and also associate with clades A, D and (particularly in the Caribbean) B [7]. Although the molecular systematics are complex and ecologically relevant units of diversity are continually being refined [8,9], the genetic markers used for identification are deemed capable of resolving reproductively isolated lineages, conventionally referred to as ‘sub-clades’ or ‘types’. Mapping the distribution of these lineages in and among corals has revealed major partitions based on geography [10,11], bathymetry [12], habitat [13–15] and, importantly, host species [16], with further divisions driven by environmental factors, such as irradiance [17], turbidity [14] and temperature [11,15]. Observed spatial partitioning and experimental manipulations have aided the formation of hypotheses regarding various physiological traits conveyed by different *Symbiodinium* types.

Here, we focus on characterizing and mapping the distribution of *Symbiodinium* assemblages hosted by the Caribbean coral *Orbicella annularis*. *Orbicella* is the dominant framework-building coral in the tropical Western Atlantic, and its growth supports diverse communities and creates important reef structure [18]. All *Orbicella* species acquire their *Symbiodinium* through open modes—from the environment—rather than inheriting them from parents. This allows the coral to be a flexible host, unlike many other Caribbean corals that show fidelity to a single symbiont taxon. *Orbicella annularis* readily associates with types from at least four genetically distinct taxa (A, B, C and D [17,19,20]), and single colonies frequently host mixed assemblages, with A/C and B/C mixes most common [16,21]. Generally, associations appear stable from year to year [22], although longer-term (decadal) shifts in community composition have been observed [23]. Most studies of *O. annularis* sampled from either one or two restricted geographical locations [14,16] and/or have only classified *Symbiodinium* to a limited genetic level [19]. More detailed information is needed to assess the relative importance of host relatedness and environmental drivers in determining *Symbiodinium* type(s) hosted by this key coral species.

## 2. Hypotheses

This study explores the hypothesis that (i) *Symbiodinium* assemblages exhibit spatial structuring in *O. annularis* across the Wider Caribbean, and (ii) structuring is driven by (H_1_) environmental heterogeneity, (H_2_) diversity within the coral host or (H_3_) geographical isolation.

### (a) Null hypothesis (H_0_): symbiont communities exhibit spatial homogeneity across the Caribbean region within *Orbicella annularis*

The current known geographical spread of symbiont types in shallow water hosts does not provide conclusive evidence for spatial patterning within *O. annularis*. *Symbiodinium* B1 is frequently the dominant symbiont in *O. annularis* colonies [7,14,17,24] and, along with less commonly occurring A-types [23], tend to be hosted by *O. annularis* in high-light environments (e.g. unshaded colony tops). Types belonging to clade C are also prevalent in this species, many of which are associated with lower-irradiance habitats, such as deeper reef areas (i.e. >10–15 m) [16,25] or the shaded sides of colonies [17,19]. As this study is concerned with the geographical rather than bathymetric distribution of *Symbiodinium*, symbiont communities may fail to exhibit any form of spatial structuring at the selected taxonomic resolution and sampling depth (approx. 6 m). Subsequently, we might expect to observe an almost uniform distribution of B1-dominated communities across the coral's range.

### (b) Alternate hypotheses (H*_X_*): symbiont communities exhibit spatial structuring across the Caribbean within *Orbicella annularis*

Previous studies have shown *O. annularis* hosting different symbiont communities across the Caribbean basin (e.g. between Panama and Belize [14], Barbados and the Mesoamerican Barrier Reef [16], and Florida and the Bahamas [24]), yet the details of how this pattern is extrapolated across the region is not known. If spatial partitioning is detected by this study, we hypothesize that observed patterns might be explained by the following.

H_1_ = *environmental heterogeneity*. Light and temperature are recognized as environmental controls on *Symbiodinium* distribution in some hosts, yet few studies examine other environmental drivers of symbiont partitioning, such as water quality, which was shown to play a role in determining symbiont assemblages hosted by a Great Barrier Reef (GBR) coral [26]. Here, we tested a suite of environmental drivers to identify those that could explain spatial partitioning.
H_2_ = *host genetics*. Spatial structuring of symbiont communities has been correlated with genetic structuring *within* their coral hosts across several sites along the GBR [12]. However, studies that have compared the population structures of Caribbean cnidarian hosts and their endosymbiotic partners have not found clear links between host and symbiont distributions, with *Symbiodinium* populations connected across geographical regions that divide the host [27].
H_3_ = *geographical distance*. Environmental and genetic factors may not adequately explain patterns in the distribution of symbionts hosted by *O. annularis*. Partitioning of clade C across the Caribbean has been shown to be a product of ecological radiations from different areas [8]. *Symbiodinium* community partitioning (if detected) may best be explained by a combination of host genetics, environmental and geographical factors, perhaps indicating reproductive isolation.

**Figure 1.**
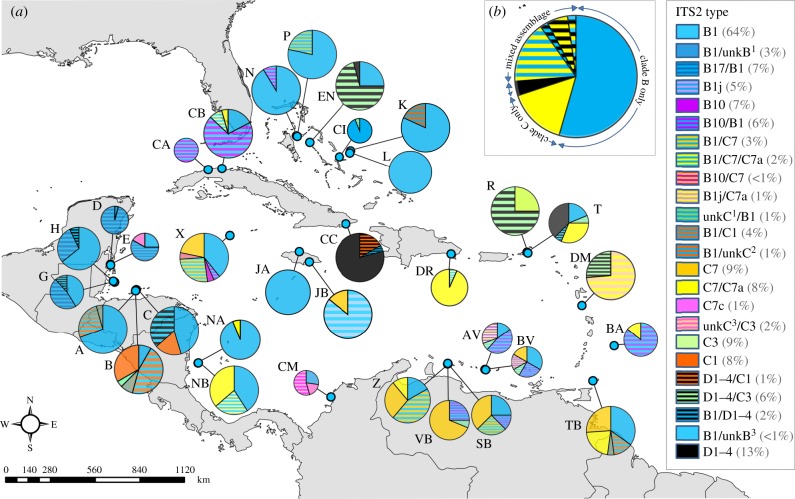
Map depicting proportions of *Symbiodinium* B (blue colours), C (yellow) and D (black) types, and combinations (stripes) hosted by *Orbicella annularis* populations at 33 sites (identified with letters; see table 1 for site information) across the Caribbean and the Bahamas. Only dominant types (or combinations of types) are represented; unk = unknown type. Pie chart size reflects colony sample size (minimum 11, maximum 24, total *n* = 632), numbers in parentheses indicate proportion of total colonies that each type was dominant in. More blue (clade B) is apparent in the northwest, with more mixed assemblages dominated by clade C (yellow) in the southeast. Inset: pie chart representing clade types found hosted by *O. annularis* for the entire Wider Caribbean area.

In addressing these hypotheses, we provide the most comprehensive dataset that exists for this species: samples originate from 33 shallow-water sites, stretching approximately 3000 km east–west from Barbados to Belize, and approximately 1800 km north–south from the Bahamas to Colombia (figure 1). By elucidating contemporary biogeographic patterns, our data contribute insights into *Symbiodinium* genetic diversity and distribution in a key Caribbean reef-building coral, while improving our understanding of the patterns of endosymbiont diversity underpinning coral reef resilience.

**Table 1.** Sampling locations, site names, sampling dates and depths, along with percentage colonies at each site that hosts either exclusively clade B, C or D types, or a mix of types, and species richness and diversity (Simpson's *D* index) for each site. ‘n.a.’ denotes data not available.

country	identifier	reef name	depth	collection date	no. samples	relative abundance of clades (%)							dominant symbiont at each site	richness	diversity
						B	C	D	B + C	B + D	C + D	B + C + D			
Honduras	A	Seaquest	4.0	Oct 2004	23	70	0	0	30	0	0	0	B1	3	0.42
	B	Sandy Bay	6.0	Oct 2004	24	17	33	0	50	0	0	0	B1/C1	6	0.20
	C	Western Wall	4.5	Oct 2004	22	82	18	0	0	0	0	0	B1	3	0.42
Belize	D	Coral Gardens	4.5	Jan 2006	24	79	0	0	0	21	0	0	B17	4	0.31
	E	Eagle Ray	2.0	Jan 2006	16	69	0	6	0	25	0	0	B1/B17	5	0.23
	G	Long Cay	6.0	Jan 2006	17	100	0	0	0	0	0	0	B1/B17	4	0.30
	H	West Reef	3.5	Jan 2006	14	100	0	0	0	0	0	0	B1/B17	4	0.29
Bahamas	CI	Conception Island	18.6	May 2007	16	94	0	0	6	0	0	0	B1	4	0.55
	EN	Exumas North	7.9	Apr 2007	24	25	0	0	0	4	50	21	D1–4	5	0.20
	K	Seahorse Reef	3.4	June 2006	22	82	0	0	18	0	0	0	B1	3	0.49
	L	Snapshot Reef	2.7	June 2006	16	100	0	0	0	0	0	0	B1	2	0.79
	N	School House Reef	3.5	June 2006	23	100	0	0	0	0	0	0	B1	3	0.50
	P	Propeller Reef	3.0	June 2006	23	78	0	0	22	0	0	0	B1	3	0.57
Nicaragua	NA	White Hole	9.0	Sep 2007	16	44	0	0	56	0	0	0	B1	5	0.22
	NB	Chavo	10.0	Sep 2007	22	18	36	0	45	0	0	0	B1/C7a	5	0.22
Colombia	CM	Palo 1	8.0	Oct 2005	11	9	73	0	18	0	0	0	C7c	4	0.27
Cuba	CA	Baracoa	4.0	Sep 2007	24	100	0	0	0	0	0	0	B10	3	0.47
	CB	Bacunayagua	4.0	Sep 2007	23	87	0	0	13	0	0	0	B10	5	0.33
	CC	Siboney	4.0	Sep 2007	24	0	0	71	0	13	17	0	D1–4	7	0.34
Cayman	X	Rum Point	5.0	Jul 2007	23	48	9	0	43	0	0	0	B1/C7	4	0.34
Dominican Republic	DR	Bayahibe	6.0	Oct 2007	15	0	93	0	7	0	0	0	C7a	5	0.29
Jamaica	JA	Drunkenmans Cay	8.0	Sep 2007	18	100	0	0	0	0	0	0	B1	3	0.68
	JB	Dairy Bull	8.0	Sep 2007	21	86	14	0	0	0	0	0	B1/B8	4	0.36
Barbados	BA	Victor's Reef	11.8	July 2007	14	86	14	0	0	0	0	0	B1j/B1	4	0.38
BVI	R	Ginger Island	n.a.	Nov 2006	24	0	4	0	0	0	79	17	D1–4	6	0.22
	T	Beef Island	n.a.	Nov 2006	16	25	31	38	6	0	0	0	B1/D1–4	6	0.14
Curaçao	SB	Snakebay	6.7	Oct 2005	16	31	38	0	31	0	0	0	C7	5	0.31
	VB	Vaersenbay	6.5	Oct 2005	16	6	69	0	25	0	0	0	C7	7	0.20
	Z	Buoy 1	4.7	Oct 2005	18	11	39	0	50	0	0	0	B1/C7	4	0.31
Dominica	DM	Grande Savane	12.0	Aug 2007	19	0	74	0	0	0	0	26	C7a	8	0.16
Tobago	TB	Buccoo Reef	3.0	Sep 2007	23	52	13	0	35	0	0	0	C1	7	0.21
Venezuela	AV	Cayo de Agua	n.a.	Aug 2007	13	38	0	0	62	0	0	0	B1j/C7a	6	0.20
	BV	Dos Mosquises	n.a.	Aug 2007	12	42	17	0	42	0	0	0	B1j/C7a	7	0.18

## 3. Methodology

*Orbicella annularis* was sampled from 33 sites across the Caribbean and the Bahamas between 2004 and 2007 (figure 1 and table 1). Single 1 cm^2^ fragments were chiselled from each of the 30 independent *O. annularis* colonies [28]. Colony tops were targeted to avoid sampling intra-colony *Symbiodinium* zonation, and collections were limited to shallow depths [17,19]. To explore symbiont diversity, a nuclear ribosomal gene, Internal Transcribed Spacer 2 (ITS2), commonly used to resolve *Symbiodinium* types [29,30], was targeted. ITS2 has some limitations in its ability to resolve (e.g. B1 [31]) and detect (e.g. D1–4 [32]) certain types, but is a widely used and well-regarded marker, appropriate for exploring broad patterns in symbiont diversity to a taxonomic level relevant to this study. Coral and symbiont DNA were extracted together using DNeasy tissue kits (Qiagen), and prevailing *Symbiodinium-*ITS2 types within each *O. annularis* individual were identified using denaturing gel gradient electrophoresis (DGGE) and direct sequencing (following [7]; see electronic supplementary material). Prominently stained gel bands (or pairs of bands) were scored as the dominant symbiont [33] against an ITS2 standard run on each gel. Representatives of every discrete, prominent band were excised under UV-transillumination, cleaned and sequenced (Macrogen) to resolve ITS2 type. Host population genetics were explored using microsatellite markers for six loci [28].

**Figure 2.**
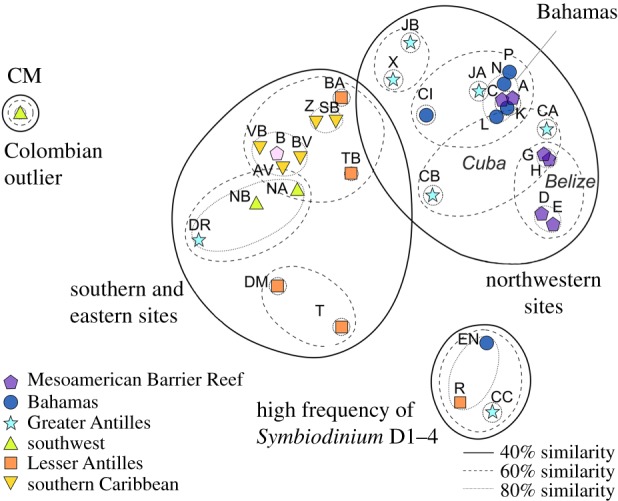
A two-dimensional MDS of square root transformed *Symbiodinium* type abundance data, based on Bray–Curtis similarities (stress = 0.12). Letters indicate site identifier code; site symbols denote ecoregion. Superimposed clusters were based on a dendrogram (not shown) of site similarities.

**Figure 3.**
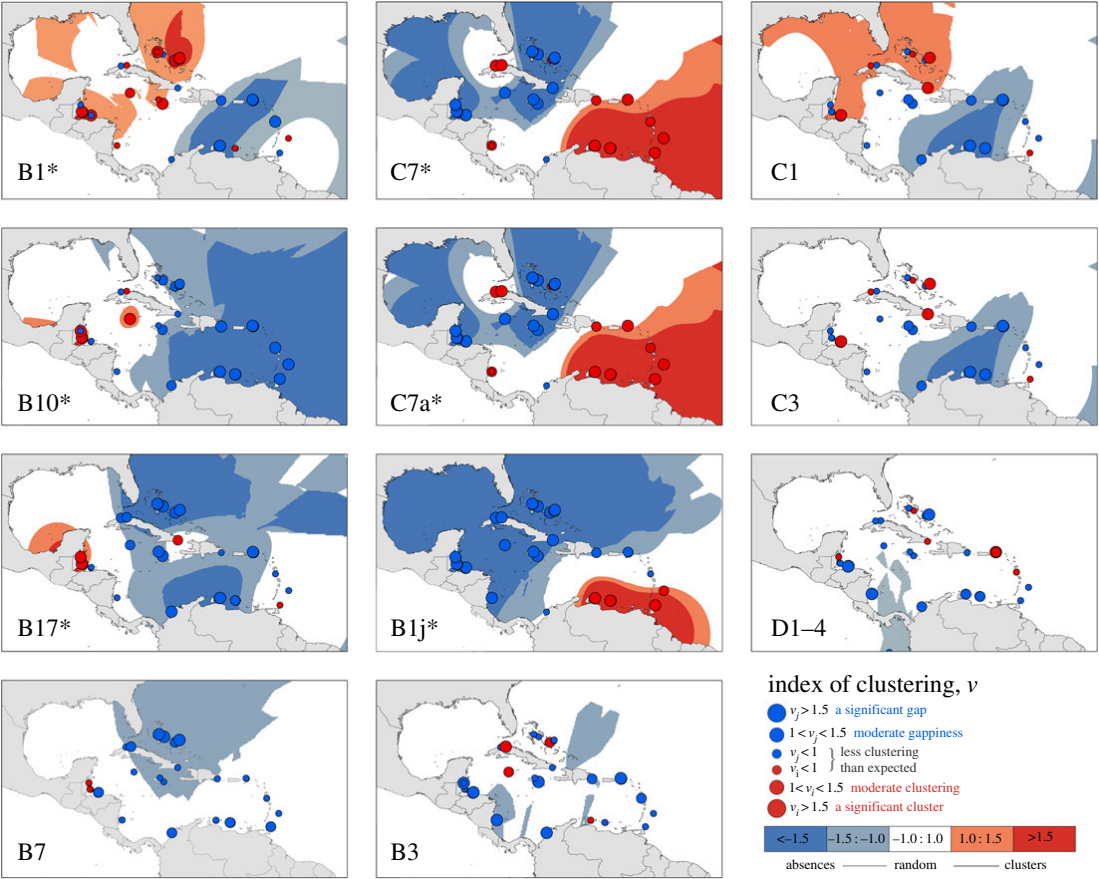
SADIE index of aggregation plots highlighting areas and sites that showed significant positive (large red circles) and negative (large blue circles) clustering in terms of the presence/absence of *Symbiodinium-*ITS2 variants. Coloured areas highlight neighbouring groups of sites that share high (or low) degrees of clustering. Six ITS2 variants (B1, B17, B10, B1j, C7 and C7a) showed significant spatial patterns; *p < 0.05.

Maps (figure 1), ordination plots (figure 2) and cluster analyses (figure 3) were used to visualize patterns in the dominant *Symbiodinium* assemblage composition across the Caribbean. To add statistical support to emergent spatial patterns, SADIE (Spatial Analysis by Distance IndicEs; a statistical approach designed for assessing the patterning of count data from spatially referenced locations) was performed on the count data for symbiont types identified [34]. SADIE generated maps showing the spatial distribution of each type (figure 3; see electronic supplementary material for details).

A distance-based linear regression analysis (DISTLM in PRIMER) was used to model the relationship between multivariate response variables (i.e. derived from representation of each *Symbiodinium* type among colonies at each site) and a suite of 15 predictors (electronic supplementary material, table S1), including environmental, geographical, temporal and host genetic variables, at the level of reef site. Marginal tests explored the amount of variability explained by each parameter considered independently. A ‘BEST’ model selection procedure was used to identify the combination of available predictor variables that best explained symbiont assemblage partitioning.

A RELATE test was used to further explore the relationship between coral host diversity and symbiont diversity at the multivariate and colony level (DISTLM targeted population level only and assessed univariate descriptors of the host). Resemblance matrices were generated for *Symbiodinium* count data and for host microsatellite allele score data for six loci (based on pairwise individual genetic distance estimated in GENALEX, 12 allele scores), for 567 individual *O. annularis* colonies [35]. The RELATE test compared the two matrices to assess the relationship between these two multivariate datasets.

## 4. Results

### (a) *Symbiodinium* assemblage diversity

The prevailing *Symbiodinium* assemblages of 632 *O. annularis* colonies were characterized for 33 sites, with an average of 18 holobiont assemblages resolved per site (figure 1). Fifteen dominant *Symbiodinium* types were identified (see electronic supplementary material, table S2 for GenBank accession numbers), nested within clades A–D. These were found in different combinations in each colony, with distinct DGGE banding profiles representing different symbiont assemblages.

Symbiont assemblages were dominated by *Symbiodinium* B1; 64% of corals sampled (405 colonies) hosted B1, 35% (221 colonies) exclusively (figure 1). B17 was the second most commonly occurring B-type, followed by B10, B1j, B3 and three unidentified types (unkB); in total, clade B was found in 70% of the corals sampled (442 colonies). Of the 70%, 80% (355 colonies) hosted *Symbiodinium* B-types exclusively, with the remainder hosting a B/C mix (72 colonies) or combinations of B/D or B/C/D (15 colonies). Almost 40% of colonies (236 colonies) hosted clade C (predominantly *Symbiodinium* C7a, C7 and C7c, and also C3 and C1, plus four–unkC–that could not be reliably identified), and 13% (83 colonies) hosted *Symbiodinium trenchii*, aka D1–4, the only known Caribbean D-type. One sample harboured A13, the only clade A type found.

### (b) *Symbiodinium* distribution patterns

Mapping the dominant symbiont type(s) revealed an emergent pattern in terms of the distribution (figure 1). Assemblages dominated by the most commonly occurring clade B-types (blue), B1, B17 and B10, showed a northwesterly distribution, while C types (yellow), particularly C7 and C7a, appeared more frequently in the eastern and southern Caribbean. D1–4-dominated assemblages were spread across six geographically dispersed sites.

Statistical analyses provided robust support to the northwest/southeast divide in the symbiont distribution identified in the mapping exercise. First, patterns of a symbiont assemblage across sites were explored using ordination plots and a cluster analysis (figure 2). Sites clustered at the 40% similarity level: southeastern populations (more diverse assemblages more often dominated by mixes of C7, C7a, B1 and B1j); central and northwestern sites (including all Cayman, Belize and Jamaican sites, all but one Bahamian and all but one Cuban site—dominated by B1); and a final group comprising three sites heavily (more than 75% of colonies) dominated by D1–4.

Geographical distance became important at the 80% level, with sites less than 100 km apart grouping, such as Belizean sites D and E (located on Caye Caulker), G and H (from Glovers Atoll), and Z and SB (neighbouring sites in Curaçao). More isolated sites (more than 100 km from any other site) hosted less similar assemblages.

SADIE analyses added further statistical support to a northwest/southeast break (figure 3; electronic supplementary material, tables S3 and S4) and were important in identifying the symbiont taxa responsible for driving this biogeographic pattern. Significant spatial patterning was identified in six symbiont taxa (electronic supplementary material, table S3): B1, B17 and B10 were more prevalent in the northwest than the southeast, while C7, C7a and B1j demonstrated the strongest partitioning (electronic supplementary material, table S3), with notable absences from most northern sites (figure 3; electronic supplementary material, table S4). No evidence of any Caribbean-wide spatial patterning was shown for D1–4 symbionts, or any other dominant type (e.g. B3, C3, C1; electronic supplementary material, table S3, figure 3). Site-specific clustering values generated by a separate cluster analysis (electronic supplementary material, table S4) confirmed that B1, B10 and B17 occurred more frequently in Belize (B17, B10) and some Honduran sites (B1), as well as most Bahamas sites (B1), but occurred less frequently than expected in easterly BVI and Curaçao sites. Meanwhile, C7, C7a and B1j were all more commonly hosted in the Lesser Antilles (southeast) and were lacking from corals across the Greater Antilles, the Bahamas and Mesoamerican Barrier Reef (figure 3; electronic supplementary material, table S4). Only one site, R, Ginger Island (BVI), reported a high cluster value for D1–4 (electronic supplementary material, table S4).

### (c) Environmental, geographical and genetic drivers of spatial patterning

Chronic thermal stress (maximum monthly mean sea temperature, °C, 1981–2010; electronic supplementary material, figure S1) was identified as the best single predictor of symbiont assemblage in marginal tests, explaining 19% of the observed variability (pseudo-*F* = 7.25, *p* < 0.001; electronic supplementary material, table S5). Both geographical distance (pseudo-*F* = 5.89, *p* < 0.001) and phosphate concentration (pseudo-*F* = 3.54, *p* = 0.011) were also identified as predictors, explaining 16% and 10% of the assemblage variation, respectively. Combining variables improved their explanatory power, with the BEST algorithm identifying a model containing seven variables (distance, acute thermal stress, chronic thermal stress, turbidity, nitrate and phosphate concentration, and sampling year) capable of explaining 54% of the variation (electronic supplementary material, table S5).

The six symbiont types (B1, B17, B1j, B10, C7a and C7) that demonstrated significant spatial patterning across the region (electronic supplementary material, table S3) were investigated independently. Negative linear relationships were identified between chronic thermal stress and the proportion of *O. annularis* colonies hosting clade C7 (*F* = 13.84, *R*^2^ = 0.41, *p* = 0.0001) and, to a lesser extent, C7a and B1j (electronic supplementary material, table S6); these three symbionts showed the strongest degree of spatial partitioning. A positive relationship between the occurrence of type B1j (the symbiont with the most strongly structured distribution, electronic supplementary material, table S3) and phosphate concentration was also identified (electronic supplementary material, table S6). B1 and B10 abundances were positively related to wave exposure, while B17 was more prevalent in turbid areas (electronic supplementary material, table S6).

Univariate metrics describing host genetics were not important in explaining symbiont assemblages in the regression analyses. Moreover, the greatest host genotypic richness was in the Bahamas—the poorest area in terms of symbiont diversity—and subsequently no association between symbiont richness and host genotypic richness was found (*F* = 0.65, *R*^2^ = 0.023, *p* = 0.322). Clonemates—*O. annularis* colonies with identical genotypes—were found only *within* sites and were dominated by the same symbiont(s) in 58% of cases (electronic supplementary material, table S7).

The RELATE analysis explored the relationship between symbiont assemblage similarity and host genetic distance matrices, and generated a Spearman's *ρ* of −0.12. This implied a weak positive relationship (*p* < 0.001) between host genetic relatedness and symbiont assemblage similarity. Repeating this analysis using different measures of symbiont assemblage (e.g. dominant symbiont types, clade level) produced similar results. When the same analysis was used to test symbiont community composition resemblance matrices against geographical distance matrices, a stronger relationship emerged (*ρ* = 0.244, *p* < 0.001).

## 5. Discussion

Mapping of symbiont distributions (supported by spatial analyses) confirmed the alternate hypotheses (H*_X_*) of spatial partitioning in *Symbiodinium* hosted by shallow water *O. annularis* across the Wider Caribbean. This was manifest as a northwest–southeast biogeographic divide, with commonly occurring types B1, B17 and B10 more prevalent in the northwest and C7, C7a and B1j in the southeast. This partitioning was best explained by chronic thermal stress (H_1_), further supported by evidence that C7, C7a and B1j tended to be absent from the sites experiencing warmer summers. Distance (H_3_) became important at smaller (less than 100 km) spatial scales, and in more fine-scale analyses. Both local environment and distance were more important than host genotype (H_2_) in explaining the symbiont assemblage, suggesting different mechanisms driving the distributions of the symbiont and its host.

### (a) Patterns of *Symbiodinium* diversity in *Orbicella annularis*

Reported Caribbean-wide distributions of endosymbiont types corroborated well with more localized previous studies on *O. annularis*. B1 has been previously reported at sites across this host's entire latitudinal range [7,14,17,33] and was the dominant *Symbiodinium* type in this study.

The distributions of other *Symbiodinium* B types (e.g. B10 in Cuba [22], B17 in Belize [16]) also supported previous studies [23] (see electronic supplementary material for details). Despite being associated with deeper/shaded colonies, types C7c, C7 and C7a occurred in reasonable abundance in our samples (agreeing with [8]), with C7 and C7a showing southeasterly distributions (figure 3). C7 has previously been reported in the western Caribbean, whereas C7a has a more easterly distribution [8,16], with overlap around Curaçao [8]. Data from the current study largely supported this (electronic supplementary material).

Not all taxa exhibited spatial partitioning: the distribution of *S. trenchii* (D1–4) was homogeneous across the sampling region with no evidence of clustering (figure 3). *Symbiodinium trenchii* made up a minor component (13%) of the total Caribbean-wide assemblages surveyed (but see [32]), and was detected only in abundance (more than 55% of colonies) at 6 of the 33 sites (dominating only three). Other studies report comparable abundances in the Caribbean, with D1–4 typically representing approximately 10% of the *Symbiodinium* community [32]. Associated with thermal stress events, the ‘patchy’ distribution of *S. trenchii* may be explained by temporary dominance at the time of sampling [25]. This is probably due to its role as an invasive opportunist [36]. Repeated sampling from these sites to test the temporal stability of the occurrence of this species would be required to explore this hypothesis.

### (b) H_1_: environmental drivers of host/symbiont biogeography

Chronic thermal stress (an indicator of routine ambient summer temperature; electronic supplementary material, figure S1) was the single most informative environmental covariate identified (electronic supplementary material, table S4), agreeing with other studies that identify temperature as a key determinant of distribution [11]. For example, where multiple environmental drivers of symbiosis were tested in *Acropora millepora* at comparable regional scales (1400 km) along the GBR [26], ‘long term’ (9-year average) SST was similarly identified as explaining the most variation in symbiont assemblage (10.8%), with summer SST and SST anomalies explaining a further 6.9% and 5.4%, respectively. Likewise, other temperature metrics remained important for explaining symbiont assemblage in this study; for example, acute thermal stress added explanatory power and was retained in most models.

An association between C7 and, to a lesser extent, C7a and B1j with chronic thermal stress was also found, suggesting that these endosymbionts may be more restricted in their distribution by thermal sensitivity than *Symbiodinium* B1 (which demonstrated a broader geographical spread), at least in *O. annularis.* An alternative explanation is that C7/C7a—usually associated with deeper *O. annularis* [16,25]—are able to tolerate living in shallower colonies only in areas where summer maxima are less extreme. Partitioning of symbiont assemblages between areas with cooler or warmer summers might influence regional bleaching performance, perhaps explaining why *O. annularis* found in cooler conditions fare worse under stress [37]. The increase in temperatures across the Caribbean due to climate change may also explain the reported replacement of C7/C7a endosymbionts by B1/B101 variants in *O. annularis* in the US Virgin Islands [23].

Phosphate concentration was also identified as a predictor, supporting Caribbean and GBR studies that identified water quality as a driver of symbiont community partitioning [14,26]. While turbidity was not an explanatory factor on its own, it was included in the best model and was found to be related to the distribution of B17 in Belize (electronic supplementary material, table S6). Meanwhile, B1j was found in greater abundance in southerly sites where phosphate levels were higher (perhaps linked to river run-off and upwelling). Phosphates are known to inhibit calcification in corals [38,39], and *Symbiodinium* are thought to remove phosphate with the addition of phosphate *and* nitrate boosting symbiont numbers [1]. However, as nitrate concentration was not found to be informative, we suggest that this pattern is more likely to be related to latitudinal gradients and river flow in the southeastern Caribbean.

### (c) H_2_: genetic drivers of host/symbiont biogeography

Host genotype could not explain symbiont community variability, leading us to reject hypothesis H_2_. No clear relationship between coral host genetic diversity and the richness of its symbiont community was apparent at the site level (DISTLM analysis), although a weak but significant association identified at the colony level (RELATE analyses) suggests an underlying environmental variable indirectly driving both factors. While breaks in the genetic structuring of *O. annularis* host populations [28] (also evident in *Acropora* sp. [40] and soft corals [27]) suggest a common biogeographic divide for many Caribbean organisms around the Mona Passage (an area of strong current flow between Puerto Rico and Hispaniola), this did not align well with symbiont distributions, with many *Symbiodinium* types present on both sides of the divide. Studies of other Caribbean cnidarian hosts also fail to identify a clear link between host and symbiont population structures, with *Symbiodinium* populations often connected across geographical features—like the Mona Passage—that divide the host [27]. For example, no association was found between the population genetics of *Symbiodinium* type C and host (*Sinularia flexibilis*) genotype on the GBR [41], or symbiont assemblages and *Acropora* spp. hosts across Micronesia [42], suggesting that different factors affect the recruitment of host and symbiont. In this study, it seems likely that the spawning nature of *O. annularis* means host and symbiont genetic diversity are not closely coupled.

### (d) H_3_: geographical distance drivers of host/symbiont biogeography

Geographical distance played a significant role in the partitioning of *Symbiodinium* community variation in this study. At the population level, the distance remained important in explaining symbiont variation in a single regression (explaining 16% of community variability). Distance was also selected as a variable in the best-fit regression model (alongside environmental parameters explaining 53% of variability). This provides support for a theory of patterns of population structure reflecting hydrodynamic circulation, as observed in clade C on the GBR [41]. While not explaining as much diversity as environmental drivers, when combined, distance parameters accounted for 20% of the symbiont assemblage variability, and were more important than the host genotype or temporal variability. Furthermore, the distance measures corresponded better with the higher-resolution symbiont community measures in the RELATE analyses. Presumably, this finding reflects the natural variation in the reservoir of free-living *Symbiodinium* available in the local environment, which in turn may reflect evolutionary radiations [8] or local environmental drivers [42].

Another explanation for the abundance of B1 in the northwest might be responses to past environmental change [43], such as marine extinctions in the western tropical Atlantic at the Pliocene/Pleistocene boundary, a hypothesis supported by the low level of ITS2 sequence divergence recorded in clade B [16,31]. Future warming of the Caribbean may even facilitate the expansion of the current range of B1 into the southern and eastern Caribbean [23]. With regions in the east (including the Dominican Republic and Curaçao) harbouring demonstrably higher-than-average community richness and diversity compared with the Bahamas, the Mesoamerican Barrier Reef and Jamaica, a gradual shift towards more B1-dominated assemblages could lessen the arsenal of available symbionts hosted within southeastern *O. annularis* populations, perhaps compromising their ability to respond to environmental disturbances.

Comparable studies that focus on symbiont biogeography have generally identified host specificity and depth zonation as key determinants of *Symbiodinium* variability [33]. The distributions described by this study are unlikely to be mirrored by other Caribbean corals, as most species demonstrate higher specificity than *O. annularis* in the formation of symbioses. Spatial diversity of symbionts in *O. annularis* is known to be greater at shallower depths (less than 8 m), and so patterns described here are unlikely to be maintained in colonies residing at deeper bathymetries, where C7 and C7a types tend to be exclusively harboured [33].

## 6. Conclusion

Identifying and mapping symbiont partitioning within a cnidarian host at this unprecedented scale is a key step in advancing our understanding of *Symbiodinium* diversity, distribution and evolution, and the potential responses of corals to future environmental change. Additionally, our results have identified a correlation between occurrences of *Symbiodinium* C7 and C7a types and milder summer temperatures, and B1j and B17 with water quality factors. Differences in *O. annularis* bleaching incidences on the Mesoamerican Barrier Reef in 1995 have been shown to be driven by the variation in the observed distribution of *Symbiodinium* communities, with fore-reef *O. annularis* undergoing a higher percentage of bleaching compared with back-reef habitats, as a greater proportion of corals hosted *Symbiodinium* C-types, which are less tolerant to stress [44]. Knowing that bleaching can vary depending on the *Symbiodinium* type(s) predominating at a site and that tolerance to bleaching roughly follows D, A>B>>C [17,25], we can extrapolate the results of the current study to predict Caribbean-wide bleaching patterns. However, it must be recognized that ITS2 markers give only a broad overview and that deeper resolution of species [31] is needed to improve the accuracy of ecological/evolutionary conclusions drawn.

The northwest–southeast Caribbean divide identified is likely to be of major consequence as corals are forced to adapt to changing environmental conditions, and may create patchiness in the severity of bleaching across the Caribbean, particularly during global bleaching events (as under way at the time of writing). Our findings on a dominant Caribbean reef-building species will help in explaining and predicting future bleaching patterns. Finally, these data provide a novel baseline that will support valuable insights into evolution-in-action in a coral species that hosts multiple symbiont assemblages.

## Supplementary Material

Kennedy et al. ESM.docx

## Data accessibility

Additional methods and description of results and four tables are included as the electronic supplementary material.
