# Supplementary material for: Symbiodinium biogeography tracks environmental patterns rather than host genetics in a key Caribbean reef-builder, Orbicella annularis
Source: Proc Biol Sci. 2016 Nov 16;283(1842):20161938. doi: 10.1098/rspb.2016.1938 (PMC5124097; doi:10.1098/rspb.2016.1938)
Supplement: Kennedy et al. ESM.docx [file rspb20161938supp1.docx]

***Symbiodinium* biogeography tracks environmental patterns rather than host genetics in a key Caribbean reef-builder, *Orbicella annularis***

Electronic Supplementary Material

Emma V. Kennedy^1,2*^, Linda Tonk^3^, Nicola L. Foster^1,4^, Iliana Chollett^5^, Juan-Carlos Ortiz^6^, Sophie Dove^3^, Ove Hoegh-Guldberg^3^, Peter J. Mumby^1,6^, Jamie R. Stevens^1^

*^1.^ College of Life and Environmental Sciences, University of Exeter, Stocker Road, Exeter EX4 4QD, UK*

*^2.^ Australian Rivers Institute, Griffith University, Nathan, 4111 Queensland, Australia*

*^3.^ Coral Reef Ecosystems Lab, School of Biological Sciences, University of Queensland, St. Lucia, 4072 Queensland, Australia*

*^4.^ School of Marine Science and Engineering, Plymouth University, Drake Circus, Plymouth PL4 8AA, UK*

*^5.^ Smithsonian Marine Station, Smithsonian Institution, Fort Pierce, 34949, FL, USA*

*^6.^ Marine Spatial Ecology Lab, School of Biological Sciences, University of Queensland, St. Lucia, 4072 Queensland, Australia*

**corresponding author:* [*emma.kennedy@griffith.edu.au*](mailto:emma.kennedy@griffith.edu.au)*; +61 (0) 7 373 57942*

***METHODOLOGY (additional information)***

*1. Collection and DNA extraction, molecular analysis.*

Denaturing gel gradient electrophoresis (‘DGGE’) and direct sequencing were used to identify *Symbiodinium* ITS2 types within each individual *O. annularis* sample [reviewed by 1]. *Symbiodinium* specific primers ‘ITSintfor2’ (5ˈ-GAATTGCAGAACTCCGTG-3ˈ) and ‘ITS2CLAMP’ (5ˈ-CGCCCGCCGCGCCCCGCGCCCGTCCCGCCGCCCCCGCCCGGGATCCATATGCTTAAGTTCAGCGGGT-3ˈ) were used to amplify a 330-360 bp product containing the ITS2 [2]; with a 12.5 µl reaction mix (1 x PCR reaction buffer, 2.5 mM MgCl_2_, 0.2 mM dNTPs, 2 U Taq DNA Polymerase, 0.6 µM primer) and PCR conditions of 95°C for 5 min; followed by 30 cycles of 94°C (45 s), 57°C (45 s) and 72°C (60 s); with a final annealing step of 59°C for 20 min. PCR products, mixed with 5 µl bromophenol blue loading buffer (15% Ficoll, 0.25% xylene cyanol FF, 0.25% bromophenol blue) were electrophoresed at 114V on a polyacrylamide denaturing gradient gel (40 to 60% denaturant) at 60°C (Ingeny System). An ITS2 standard (with B1, C1, and C3; provided by the Coral Reef Ecosystems Lab, University of Queensland, Australia) was run in the first lane of each gel. After 14 h, the gel was stained with SybrGreen I (Invitrogen) nucleic acid gel stain at room temperature for 20 min, before imaging in a UV transilluminator. Imaged gels were examined by eye and scored for types, with comparison to a database of other gels used to help identify haplotypes. Dominant bands from each DGGE gel were excised, cleaned and sent for sequencing (Macrogen) to resolve ITS2 type.

Imaged gels were examined carefully by eye. DGGE produces diagnostic fingerprints or ‘profiles’, consisting of high-melting-lower-migrating homoduplexes, and low-melting-higher-migrating-heteroduplexes. When two or more symbionts are abundant in a sample [> 10% of the population; 3], the fingerprint profiles of each are identifiable in the same lane [4]. The more prominently stained homoduplex band (or bands), was scored as the dominant symbiont [5]. Representatives of every discreet, prominent band were excised under UV-transillumination and stored at 4°C overnight in 30 µl RNAse-free water. Reamplification was performed with 1 µl eluate, using the primers ITSinfor2 and ITS2-reverse (5ˈ-GGGATCCATATGCTTAAGTTCAGCGGGT-3ˈ). Exonuclease 1 and Antarctic phosphatase were used to clean 2 µl of the PCR product (37°C for 15 min and a further 15 min at 80°C), and samples diluted to 6-12 ng µl^-1^ for sequencing. The product was sequenced in both directions using both forward and reverse amplification primers separately (Macrogen). A sequence alignment was performed in Clustal X and checked by eye, prior to comparison against a database of all known Caribbean *Symbiodinium* types in the Geosymbio database [6].

*2. Spatial analyses*

After scoring, data on *Symbiodinium* distribution was superimposed onto a map (Fig. 1). To explore patterns of symbiont distribution across sites, a Bray Curtis similarity matrix was constructed based on root-transformed *Symbiodinium* type data, and ordination plots and a cluster analysis were used to visualise similarities and differences in the dominant *Symbiodinium* assemblage composition across the Caribbean (Fig. 2). To further determine the significance of apparent spatial patterns, SADIE (Spatial Analysis by Distance IndicEs; a statistical approach designed for assessing the patterning of count data from spatially referenced locations) was used to separately analyse geographic patterns in the distribution of all ITS2 types [7]. The analysis was run on spatially referenced count data, separately for each of the major *Symbiodinium* types identified, with 5796 randomizations and parameters (*iseed* 30,000; k5psim=153; 5796 randomisations) (Table S1). SADIE assesses the significance of spatial patterns using an index of clustering based on geographic distance: it assigns each site either a positive patch cluster (*v_i_*) or a negative gap cluster (*v_j_*) value, based on whether abundance of any given ITS2 type is higher or lower than expected by chance (Table S2). These *v* values were then plotted onto ‘class-post’ maps (with a contouring interval values of 1.5 and -1.5, chosen to reflect clustering that is half as large again as expected from a random arrangement of the counts) and universal kriging used to interpolate between the data points to produce a Red-Blue Plot indicating clustering of spatial data [8] (Fig. 3). Red areas on the map indicate ‘patch clusters’ (spatial areas where any given symbiont type is more abundant than expected by chance), while blue represents ‘gaps’ (areas where there is a significant scarcity of any given symbiont compared to abundance values expected by chance).

*3. Environmental, geographic and genetic predictors*

Colony-level data on the presence/absence of symbiont taxa, and population-level data on the relative proportions of colonies hosting different clades per reef site, were used to investigate the broad-scale partitioning in host-symbiont associations. A suite of twenty-three environmental, geographic, genetic and temporal predictors were determined for each reef site and included a combination of genetic data (based on six polymorphic microsatellite loci describing the genetic structure of the *O. annularis* host: [9, 10], remote sensing data and environmental data gathered during sample collection, geographic determinants based on the reef location and information on month and year of sampling. After discarding eight collinear covariates to avoid type II errors, just 15 explanatory variables remained (Table 2). Variables were then transformed (square root transformations were applied to *Turbidity*, and a log (*c*+*y*) transform applied to *Acute thermal stress (severe)*, *Enclosure* and other SST variables), and a final inspection of draftsman plots based on the retained, transformed variables revealed satisfactory fitted assumptions. Data on symbiont abundances were first pooled and standardised (to account for difference in sample sizes) to give a percentage presence/absence for each site. Rare symbionts that were found only at one site were removed from the dataset, as the statistical multiple linear regression method can be sensitive to low abundances, although later comparisons showed that this made little difference to outcomes. Symbiont abundances were root transformed to down-weight the importance of heavily dominant B1, Bray-Curtis resemblance matrices were generated and ordination plots were produced to further explore the data.

A distance-based linear regression (DISTLM) was used to model the relationship between our multivariate response variables (e.g., communities of symbionts) and our predictors. DISTLM analyses were performed at the level of reef site, because most environmental data were available at this resolution (1-4 km resolution). The DISTLM regression analysis was performed using an add-on PERMANOVA+ in software package PRIMER (PRIMER-E Ltd, Plymouth Marine Laboratory). Unlike most regression models, p-values are obtained through permutation, avoiding the usual assumptions that errors are normally distributed. The final model contained 15 unrelated variables, including environmental and temporal (Year) (Table 2). Marginal tests explored the amount of variability explained by each parameter considered independently.

In order to identify the combination of available predictor variables that best explained symbiont community partitioning, we employed a ‘BEST’ model selection procedure, which examines the value of the selection criterion for ALL possible combinations of variables. The ten most informative models (Table S3) were selected on the basis of AIC (Akaike Information Criterion).

A RELATE statistical test (PRIMER-E Ltd, Plymouth Marine Laboratory) was used to explore the explicit relationship between coral host diversity and symbiont community diversity at the colony level (i.e. at a higher resolution). RELATE produces a measure of how closely related two datasets are, with probabilities based on the number of permuted statistics greater than or equal to Spearman’s ρ. A ρ value close to 1 indicates that the matrices are more similar/have a similar distribution, a ρ value closer to 0 indicates a weak relationship between the matrices. Two multivariate datasets for a matching set of samples: one containing pairs of genetic distance scores for the six microsatellite loci (12 allele scores, 567 individuals) belonging to the host colony, and one describing the presence/absence of each *Symbiodinium* sub-cladal ITS2-type for the same colony’s endosymbiont community, were compared using Spearman’s Rank correlation coefficients. This was done by first generating two resemblance matrices, using a Sorenson coefficient to generate a matrix for symbiont count data (18 variables, 567 samples) and a matrix based on pair-wise individual genetic distance (estimated in GENALEX for the microsatellite allele score data (12 variables, 567 samples). Variation between elements in the first matrix were then compared to those in the second by calculating a rank correlation coefficient (Spearman’s ρ) of the two matrices, and the coefficient value compared to 9999 permutations of matrix data (significance level of sample statistic: 0.01%), using PRIMER.

***DISCUSSION (additional information)***

B1, a type known to be common across multiple Caribbean hosts [4, 11], was identified as the dominant *Symbiodinium* type hosted by *O. annularis* in this study. Given that B1 has been previously reported at sites across *O. annularis*’s entire latitudinal range [4, 5, 14-17] – particularly at shallower depths – it is unsurprising that it was identified so frequently in this study: localised sampling in previous studies having lacked the coverage required to effectively map distribution range within this species. B1’s ecological dominance in the Caribbean has been attributed to it being a generalist species [12], and its presence attributed to adaptive radiation in the Caribbean [13]. Substantial cryptic diversity is suspected to exist within ITS2 B1, with at least three (maybe four) distinct lineages, analogous to species, existing within the sub-clade [11, 18-20], which have been demonstrated to show biogeographic partitioning in the sea fan *Gorgonia ventalina* [21]. Selection of ITS2 as a molecular marker may have caused important functional diversity within B1to be overlooked.

The occurrence of other B type endosymbionts (e.g., B10, B17) in the *O. annularis* populations studied also corroborates well with previous studies [22]. B10 was most abundant in northern Cuba (samples CA and CB) – just 320 km from where it was reported in populations at Little Grecian Reef in Florida [23], but was absent from eastern and southern Caribbean regions. B17, a type found only in *O. annularis* specifically from the western Caribbean [17] - dominated symbiont assemblages at Belizean sites, with a few cryptic occurrences in Cuba and Tobago. This supports another study which isolated B17 from *Orbicella* in Belize, but not in Barbados: microsatellite work has suggested this endosymbiont type to be very closely related to B1 in *O. annularis* [11]. B1j was the final B type to show significant spatial structuring: the pattern of distribution was the inverse of B1, B17 and B10, with significant clustering revealed across the southern Caribbean. B1j has been documented previously in this area [11, 24], as well as in the eastern Caribbean. B1j was spatially associated with C7 (co-occurring 40% of the time) and C7a (28%), showing a 40% similarity in their distribution. In two Barbados studies [11, 24], shallow (6-10 m) colonies contained a B1/C7a or B1j (high light)/ C7a mix. B1j was also found to replace *Symbiodinium* *trenchii* after bleaching on shallow Barbados reefs [24], and its prevalence in this location might be caused by the bleaching that occurred in the eastern Caribbean prior to this study [25].

Some *Symbiodinium* C types can sometimes be associated with deeper/shaded colonies (e.g., C7a documented in *O. annularis* colonies at >10-15 m in Barbados [11, 24]), yet types C3, C7 and C7a were found in reasonable abundance in shallow water colonies in this study; of these variants, only C7 and C7a demonstrated significant spatial structuring. Finney *et al*. [11] observed that deeper *O. annularis* species complexes from Barbados hosted C7a, while their counterparts in the western Caribbean (Belize) hosted C7 instead, suggesting analogous east/west symbionts with similar ecological traits. Kemp et al. 2008 also found C7 in another *Orbicella* species in Florida at 2 m [34]. However, a difference in the distribution of C7 and C7a was not apparent in the current study, with both types found more frequently in the east than the west (Fig. 1), although the two Cuban sites (CA and CB) closest to Florida hosted a moderate amount of C7a too (Fig 3, Table S2). This may be because the study of Finney *et al.* used six host coral species and included *O. franski* and *O. faveolata* (which appeared to drive trends in their data). C7 has previously been described in the western Caribbean, while C7a has a more easterly distribution [11, 26], with overlap in the distributions around Curaçao [26]. Data from the current study largely supported this: *Symbiodinium* from Barbados and the BVI showed more C7a, and their distributions overlapped with C7a around Curaçao.

*Symbiodinium* type A13 (also known as A1.1), thought to be a highly opportunistic symbiont, was only found in one sample. A13 is ecologically rare: one study identified only four clade A *Symbiodinium* in over 476 Caribbean cnidarians [11]; another study only observed clade A once in very shallow (0-3 m) back reef habitats [4, 14]; a third demonstrated clade A associated with *O. annularis* only in severely bleached individuals [24]. As bleached and very shallow colonies were not sampled, this might explain why the type was detected just once in the current study.

*Symbiodinium* *trenchii*, a.k.a. D1-4 and D1a, was recorded in abundance (>55% of colonies) at just 6 of the 33 sites, but its distribution was shown to be homogenous across the sampling region with no evidence of spatial patterning (Fig. 3). At three of these locations – the Exumas (Bahamas, EN), Ginger Island (BVI, R) southeast Cuba (CC) – *S.* *trenchii* was found in 75%, 96% and 100% of samples, respectively, yet neighbouring sites rarely harboured *Symbiodinium* D types. *Symbiodinium* *trenchii* is known to have a stress-tolerance that exceeds that of B and C types, allowing it to proliferate in corals experiencing sub-optimal reef conditions, usually in terms of higher than average thermal stress, but in the case of *O. annularis*, also in turbid environments [30] and habitats affected by sedimentation [14, 31]. *Symbiodinium* *trenchii* also appears in *O. annularis* in the build-up to bleaching events [24] and, although *O. annularis* typically revert to pre-bleaching symbiont assemblages [32], after severe bleaching events this can take 2-3 years [30].

Both British Virgin Islands sites hosted *Symbiodinium* assemblages that were heavily dominated by *S.* *trenchii*, but Ginger Island was the only site to generate a significant value of clustering (v=1.93; Table S3, Fig. 3). This may be due to bleaching at these sites prior to sampling [33]. Records show that at the time of sampling this site (November 2006), coral communities in the region were still in the process of recovering from the 2005 bleaching event, with partial colony bleaching still prevalent [33]. Tobago and Barbados were also affected by the 2005 bleaching event, but did not harbour *S.* *trenchii* profiles. As both these sites were sampled in 2007, *Symbiodinium* assemblages may have had time to recover to pre-bleaching symbiont assemblages. It was also noted that in Tobago, most bleaching occurred at deeper sites [33], whereas sampling took place at 3 m. Finally, the patchy nature of *O. annularis* bleaching recorded in Barbados [33] may have provided researchers with more opportunities to select healthy looking colonies for sampling. Given the “patchy” nature of the occurrences of *S.* *trenchii*, exposure to a thermal stress event might be a more likely explanation for its apparently random occurrence. The ‘patchy’ distribution of *S.* *trenchii* may be explained by the hypothesis that a substantial proportion of corals harbour cryptic clade D that later become temporarily dominated by *S. trenchii* during or after a stress event [24]. This is likely due to its role as an invasive opportunist [28]. It would be interesting to revisit these sites post-bleaching to see if the amount of detectable *S. trenchii* subsequently increases.

**Supplementary figures**

**
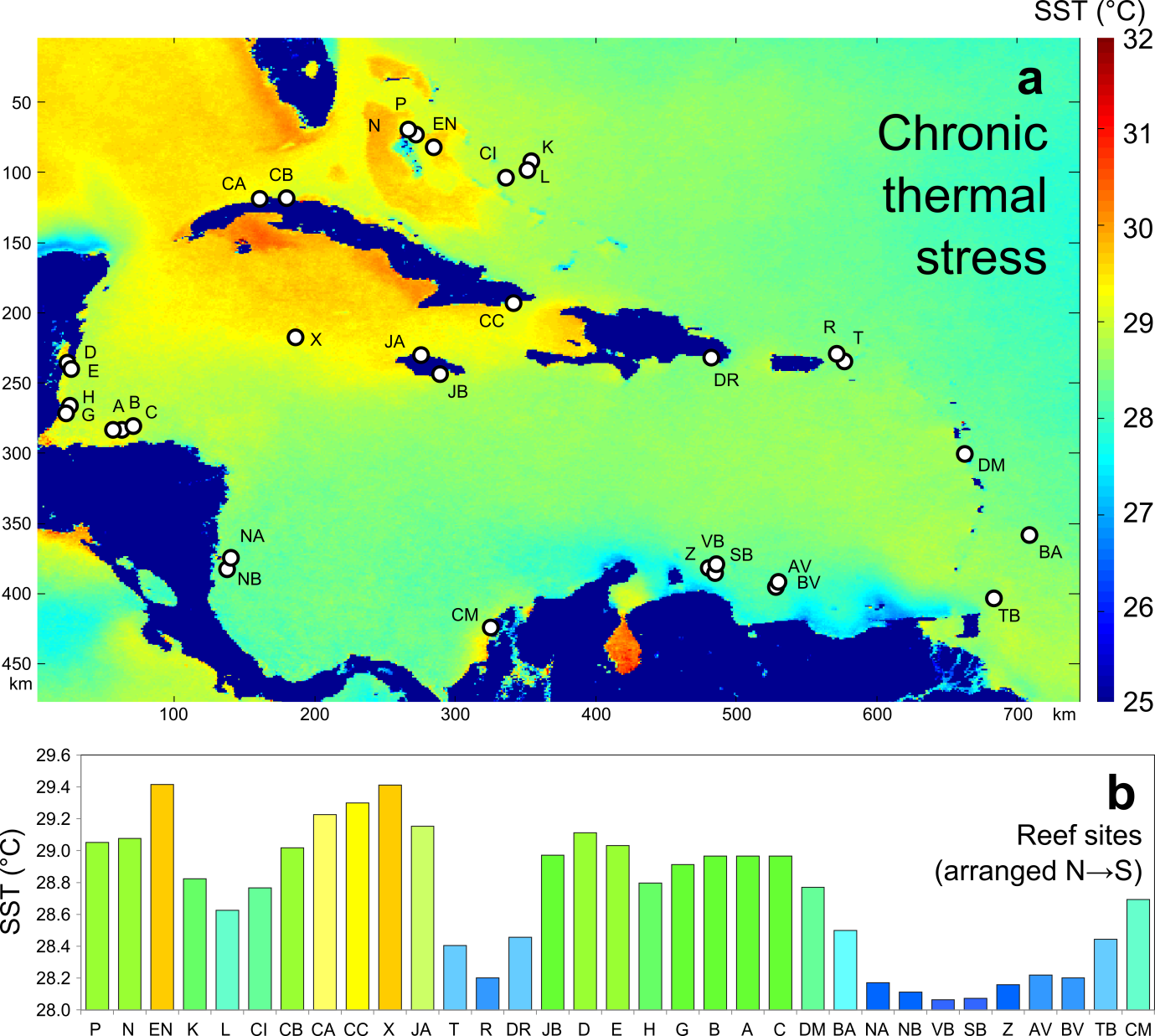
**

**Figure S1**. Thermal stress metrics used to inform the statistical model **a)** Chronic thermal stress shown by maximum monthly mean sea surface temperatures (1981-2010) from the AVHRR Pathfinder dataset. White circles represent sampling sites (for identifiers see Table 1). **b)** Bar chart displaying the chronic thermal stress value (°C) for each of the reef sites arranged by latitude.

**Supplementary tables**

**Table S1**: List of the geographic, environmental, genetic and temporal parameters included in the final model designed to explain *O. annularis* symbiont biogeography. Initial model contained 23 covariates, eight were discarded after testing which revealed collinearity. Final four columns show summary of DISTLM population-scale marginal tests, examining the relationship between *Symbiodinium* assemblages and explanatory covariates. Significant variables highlighted in blue. ‘SS(trace)’ = sums of squared deviations (partitioning of variance), ‘Pseudo-F’ = test statistic, ‘P’=interpretation of test stat (p-value). ‘Prop.’= proportion of variance explained by model term. Residual df=30.

| **Variable** | **Description** | **Units** | **Source** | **SS(trace)** | **Pseudo-F** | **P** | **Prop.** |
| --- | --- | --- | --- | --- | --- | --- | --- |
| Geographic proximity | *PCO1 estimate of the resemblance (derived from lat-long data)* | none | This study | 9356 | 5.89 | 0.0009 | **0.16** |
| Acute thermal stress | *Number of DHW above zero, for the year prior to sampling (4 km resolution)* | frequency | AVHRR pathfinder v5.2, this study | 1358.7 | 0.73 | 0.5744 | <0.1 |
| Acute thermal stress (severe) | *Number of DHW above four, for the year prior to sampling (4 km resolution)* | frequency | AVHRR pathfinder v5.2, this study | 2311.1 | 1.27 | 0.2703 | <0.1 |
| Chronic thermal stress | *Maximum of monthly mean SST for all years in record (1981-2010) (4 km resolution)* | °C | AVHRR pathfinder v5.2, this study | 11106 | 7.25 | 0.0001 | **0.19** |
| History of thermal stress | *Number of DHW above zero from 1981 until one year prior to sampling (4 km resolution)* | frequency | AVHRR pathfinder v5.2, this study | 3996 | 2.26 | 0.0651 | <0.1 |
| Turbidity | *Average chlorophyll-a concentration during the year of sampling (4 km resolution)* | mg m^3^ | MODIS daily Chlor_a, this study | 3821.6 | 2.15 | 0.0775 | <0.1 |
| Salinity | *Climatological average of surface salinity* (0.25° resolution) | PSS | World Ocean Atlas 2009, this study | 2846.7 | 1.58 | 0.1655 | <0.1 |
| Nitrate concentration | *Climatological average of surface nitrate concentration* (1° resolution) | μ mol l^-1^ | World Ocean Atlas 2009, this study | 3118.5 | 1.74 | 0.1336 | <0.1 |
| Phosphate concentration | *Climatological average of surface phosphate concentration* (1° resolution) | μ mol l^-1^ | World Ocean Atlas 2009, this study | 6028.3 | 3.55 | 0.0114 | **0.11** |
| Wave exposure | *Natural log of the climatological average of wave exposure (1 km resolution)* | Ln(j m^-3^) | Chollett *et al*. 2012 [63] | 2783.7 | 1.54 | 0.1873 | <0.1 |
| Enclosure | *Reciprocal of the sum of the distance to land in cardinal directions (0.1 km resolution)* | 1/m | Adapted from Garren *et al*. 2006 [23] | 2302.5 | 1.26 | 0.1773 | <0.1 |
| Depth | *Mean depth of the reef site* | m | Foster 2007 [71]. | 3353.7 | 1.87 | 0.1147 | <0.1 |
| Host genotypic diversity | *Clonal diversity: number of genotypes/number of samples* | frequency | Foster *et al*. 2012 [42] | 647.59 | 0.34 | 0.8978 | <0.1 |
| Host heterozygosity (HE) | *Genetic variation in a population, based on the squared allele frequencies* |  | This study / Foster 2007 [71]. | 526.72 | 0.28 | 0.9168 | <0.1 |
| Sampling year | *Year (2003-2007) that sampling took place* | 1 year | Foster 2007 [71]. | 1402.2 | 0.76 | 0.5588 | <0.1 |

**Table S2**: All *Symbiodinium* ITS2 types identified by DGGE analysis in this study, along with GenBank accession numbers and details on sampling locations from this and previous studies. In some cases only one or two examples of these were found so not included in analyses.

| **Clade** | **ITS2 type** | **Genbank no.** | **Detected in**  ***O. annularis* from** | **Previously reported in**  ***O. annularis*** | **References** |
| --- | --- | --- | --- | --- | --- |
| Clade A | A13 (A1.1) | AF333504 | Barbados | Barbados | LaJeunesse 2009 |
| Clade B | B1 | AF333511 | Bahamas; Barbados; Belize; Cayman Islands; Columbia; Cuba; Curaçao; Dominican Rep.; Honduras; Jamaica; Nicaragua; Tobago; US Virgin Islands; Venezuela | Exuma Islands & Florida Keys; Barbados; Bahamas; US Virgin Islands; Bermuda | Thornhill, 2009, LaJeunesse, 2009 & Finney 2010, LaJeunesse, 2002, Correa, 2009, Savage 2002 |
|  | B3 |  |  |  |  |
|  | B10 | AF499787 | Cayman Islands; Cuba | Florida Keys | Thornhill, 2009 |
|  | B17 | AY074987 | Belize | n/a | Savage, 2002 |
|  | B1j | GU907637 | Barbados; Belize; Curaçao; Venezuela | Eastern Caribbean | Finney, 2010, LaJeunesse 2009 |
| Clade C | C1 | AF333515 | Bahamas; Cuba; Honduras; Tobago | US Virgin Islands | Correa, 2009 |
|  | C3 | AF499789 | Belize; Columbia; Dominica | US Virgin Islands; Exuma Islands; Florida Keys | Correa, 2009, LaJeunesse, 2002, Thornhill, 2006, 2009 |
|  | C7 | AF499797 | Bahamas; Barbados; Cayman Islands; Cuba; Curaçao; Dominica; Dominican Rep.; Jamaica; Nicaragua; US Virgin Islands; Tobago; Venezuela | US Virgin Islands; Barbados | Correa, 2009, Finney, 2010,  LaJeunesse, 2009 |
|  | C7a  (C12) | AF499801 | Bahamas; Barbados; Cuba; Curaçao; Dominica; Dominican Rep.; Nicaragua; US Virgin Islands; Tobago; Venezuela | Eastern Caribbean; Exhuma Islands | Finney, 2010, LaJeunesse, 2009, Thornhill, 2009 |
|  | C7c |  |  |  |  |
| Clade D | *Symbiodinium trenchii*  (D1-4, D1a) | AF499802 | Bahamas; Belize; Cuba; Dominica; Honduras; US Virgin Islands | Exuma Islands; US Virgin Islands; South Yucatan; Florida Keys | LaJeunesse, 2002, Correa, 2009, Finney, 2010, LaJeunesse, 2009, Thornhill, 2006 |

**Table S3**: SADIE outputs, describing the indices for evaluating the spatial distribution of each observed *Symbiodinium* ITS2 type. Cells highlighted pink indicate significant values.

| Symbiont  type | Mean | Variance | Index of dispersion | δ | Distance to Regularity, D | | Clustering indices | | | |
| --- | --- | --- | --- | --- | --- | --- | --- | --- | --- | --- |
|  |  |  |  |  | *I_a_* | *P_a_* | *v_i_* | | *v_j_* | |
|  |  |  |  |  | Index of aggregation | Significance of Ia | Patch cluster index | | Gap cluster index | |
|  |  |  |  |  |  |  | mean | p-value | mean | p-value |
| B1 | 34 | 363.6 | 342.2 | 1.91 | 1.72 | 0.02 | 1.78 | 0.02 | -1.51 | 0.08 |
| B17 | 34 | 169.3 | 159.3 | 1.08 | 1.63 | 0.03 | 2.15 | 0.01 | -1.67 | 0.04 |
| B1j | 34 | 191.2 | 179.9 | 2.36 | 2.75 | 0.00 | 2.91 | 0.00 | -3.21 | 0.00 |
| B10 | 34 | 145.6 | 137.1 | 1.41 | 1.86 | 0.00 | 2.51 | 0.00 | -1.72 | 0.03 |
| C1 | 34 | 211.5 | 199.1 | 0.73 | 1.15 | 0.25 | 1.25 | 0.18 | -1.08 | 0.34 |
| C (unk) | 34 | 33.0 | 31.1 | 0.23 | 0.91 | 0.73 | 1.00 | 0.93 | -0.87 | 0.72 |
| C7c | 34 | 211.0 | 199.1 | 0.73 | 1.15 | 0.25 | 1.25 | 0.18 | -1.08 | 0.34 |
| C7 | 34 | 338.9 | 319.0 | 2.59 | 2.29 | 0.00 | 2.05 | 0.01 | -2.93 | 0.00 |
| C7a | 34 | 322.8 | 303.8 | 2.54 | 2.30 | 0.00 | 2.43 | 0.00 | -2.74 | 0.00 |
| B7? | 34 | 93.1 | 87.6 | 0.22 | 1.02 | 0.44 | 0.90 | 0.59 | -1.01 | 0.46 |
| B3? | 34 | 145.6 | 137.1 | 0.64 | 1.15 | 0.25 | 1.12 | 0.28 | -1.18 | 0.23 |
| C3 | 34 | 93.1 | 87.6 | 0.75 | 1.36 | 0.09 | 1.25 | 0.15 | -1.43 | 0.08 |
| D1-4 | 34 | 145.6 | 137.1 | 1.11 | 1.02 | 0.40 | 1.37 | 0.13 | -1.78 | 0.03 |
| A13 | 32 | 33.0 | 31.1 | 0.49 | 1.45 | 0.08 | 1.01 | 0.06 | -1.52 | 0.00 |

**Table S4**: Cluster indices generated by SADIE analysis for symbionts at each site. Blue cells indicate values with a significant v_j_ value (demonstrating negative clustering), while red cells (significant v_i_) depict sites where symbiont appeared to be more clustered than would occur by random chance (measured against 5000+ random permutations).

| Area | Country | Reef | Site | Latitude | Longitude | A13 | B1 | B10 | B17 | B1j | B3 | B7 | C1 | C7a | C3 | unkC | C7 | C7c | D1a |
| --- | --- | --- | --- | --- | --- | --- | --- | --- | --- | --- | --- | --- | --- | --- | --- | --- | --- | --- | --- |
| Meso-american Barrier Reef | Honduras | Seaquest | A | 16.2940 | -86.6000 | -2.18 | 4.39 | -0.30 | -0.32 | -4.77 | -0.01 | -0.99 | 0.42 | -1.51 | 0.42 | -0.96 | -4.25 | -3.61 | -3.96 |
|  | Honduras | Sandy Bay | B | 16.3340 | -86.5680 | -2.15 | 0.00 | -0.27 | -0.26 | -3.78 | 0.20 | -1.00 | 2.13 | -2.08 | 2.13 | -1.01 | -3.56 | -3.63 | -2.55 |
|  | Honduras | Western Wall | C | 16.2710 | -86.6040 | -2.16 | -0.39 | -0.30 | -0.31 | -4.41 | -0.02 | -0.96 | 2.01 | 0.01 | 2.01 | -1.01 | 0.00 | -3.51 | -5.15 |
|  | Belize | Coral Gardens | D | 17.7484 | -88.0233 | -2.17 | -0.12 | -0.01 | 2.27 | -4.92 | -1.78 | -1.07 | -0.51 | -2.39 | -0.51 | -0.94 | -2.38 | -2.04 | -2.69 |
|  | Belize | Eagle Ray | E | 17.7203 | -88.0136 | -2.11 | 0.01 | 3.87 | 2.69 | -5.43 | -0.44 | -1.08 | -0.62 | -6.81 | -0.62 | -0.95 | -6.52 | -2.09 | -2.97 |
|  | Belize | Long Cay | G | 16.7545 | -87.7861 | -2.12 | 4.82 | 1.53 | 1.66 | -6.04 | -0.32 | -1.08 | -0.41 | -2.35 | -0.41 | -0.99 | -2.29 | -2.11 | -3.55 |
|  | Belize | West Reef | H | 16.8088 | -87.8621 | -2.15 | 2.86 | 4.50 | 4.37 | -6.02 | -0.35 | -1.09 | -0.45 | -2.59 | -0.45 | -1.01 | -2.43 | -2.12 | -3.55 |
| The Bahamas | Bahamas | Conception Island | CI | 23.8120 | -75.1218 | -1.77 | 2.44 | -1.35 | -2.92 | -3.90 | -2.85 | -0.91 | -0.39 | 0.45 | -0.39 | -0.25 | 0.47 | -0.28 | -2.41 |
|  | Bahamas | Exumas North | EN | 24.6409 | -76.7954 | -1.88 | -0.24 | -1.13 | -3.18 | -5.13 | -3.45 | -0.99 | 0.78 | -3.14 | 0.78 | -0.08 | -2.55 | 1.08 | 1.31 |
|  | Bahamas | Seahorse Reef | K | 24.1582 | -74.4839 | -1.67 | 2.67 | -1.32 | -3.05 | -3.83 | -2.63 | -0.95 | 1.73 | -2.97 | 1.73 | -0.29 | -3.27 | -1.63 | -2.09 |
|  | Bahamas | Snapshot Reef | L | 24.0314 | -74.5297 | -1.71 | 2.95 | -1.34 | -1.48 | -3.79 | -2.62 | -0.93 | -0.03 | -4.01 | -0.03 | -0.29 | -3.08 | -1.64 | -2.09 |
|  | Bahamas | School House Reef | N | 24.9734 | -77.5051 | -1.87 | 3.01 | -0.98 | -3.03 | -4.18 | -3.51 | -1.02 | -0.22 | -3.94 | -0.22 | -0.01 | -6.24 | -0.11 | -0.16 |
|  | Bahamas | Propeller Reef | P | 25.0064 | -77.5524 | -1.91 | 0.95 | -0.96 | -2.73 | -3.96 | -1.75 | -1.02 | -0.23 | -3.66 | -0.23 | 1.00 | -5.75 | -0.12 | -0.17 |
| Nicaragua/Columvbia | Nicaragua | White Hole | NA | 12.1881 | -83.0518 | -1.91 | 0.83 | -0.80 | -0.87 | -1.97 | -1.69 | -0.92 | -0.78 | 0.92 | -0.78 | -1.14 | 1.13 | -2.40 | -2.34 |
|  | Nicaragua | Chavo | NB | 12.1835 | -83.0670 | -1.89 | 0.00 | -0.82 | -0.90 | -2.13 | -1.75 | -0.94 | -0.87 | 1.17 | -0.87 | -1.14 | 1.19 | -2.67 | -2.41 |
|  | Columbia | Palo 1 | CM | 10.2770 | -75.6110 | -1.34 | -0.94 | -1.45 | -1.50 | -0.97 | -0.72 | 0.96 | -1.38 | -0.95 | -1.38 | -1.22 | -0.73 | -1.46 | -1.45 |
| Greater Antillies+ Cayman | Cuba | Baracoa | CA | 23.0871 | -82.5077 | -2.10 | -0.88 | -0.72 | -1.24 | -3.35 | -1.00 | -0.56 | 0.82 | 1.84 | 0.82 | -0.45 | -0.18 | -0.83 | -0.86 |
|  | Cuba | Bacunayagua | CB | 23.1507 | -81.7274 | -2.09 | 0.17 | 0.83 | -1.39 | -3.42 | -1.01 | -0.53 | -0.14 | 2.03 | -0.14 | -0.40 | 1.13 | -0.73 | -0.82 |
|  | Cuba | Siboney | CC | 20.0315 | -74.7548 | -1.73 | -0.59 | -0.89 | 1.22 | -1.56 | -1.31 | -0.40 | 1.51 | -2.38 | 1.51 | -0.59 | -1.58 | -1.40 | 1.35 |
|  | Cayman | Rum Point | X | 19.3776 | -81.2810 | -2.20 | 1.22 | 1.80 | -1.03 | -2.22 | -0.58 | 0.88 | -0.85 | -2.35 | -0.85 | -0.66 | 1.22 | -0.98 | -0.96 |
|  | Dominican Republic | Bayahibe | DR | 18.3440 | -68.8314 | -0.93 | -1.16 | -1.57 | -0.80 | -1.37 | -0.83 | -0.98 | -1.18 | 1.21 | -1.18 | -0.98 | 1.06 | -0.52 | -0.53 |
|  | Jamaica | Drunkenmans Cay | JA | 18.4688 | -77.3856 | -1.97 | 0.90 | -0.75 | -1.57 | -1.91 | 0.80 | -0.11 | -1.11 | -2.79 | -1.11 | -0.69 | -1.16 | -1.68 | -0.43 |
|  | Jamaica | Dairy Bull | JB | 17.8876 | -76.8288 | -1.88 | 1.65 | -1.50 | -1.53 | -1.69 | -0.11 | 0.86 | -1.43 | -1.76 | -1.43 | -0.76 | 0.14 | -1.56 | -1.64 |
| Lesser Antillies | Barbados | Victor's Reef | BA | 13.1630 | -59.6409 | 1.01 | 0.52 | -3.43 | -0.30 | 1.36 | -0.89 | -1.50 | -0.36 | 3.84 | -0.36 | -1.20 | 3.79 | -0.27 | -0.34 |
|  | BVI | Ginger Island | R | 18.3915 | -64.4838 | -0.51 | -2.27 | -2.26 | -1.56 | -1.13 | -0.96 | -1.50 | -1.95 | -0.92 | -1.95 | -1.05 | -0.87 | 1.60 | 1.93 |
|  | BVI | Beef Island | T | 18.4395 | -64.5351 | -0.52 | -2.15 | -2.22 | -1.53 | -1.06 | -0.95 | -1.48 | -1.87 | 2.29 | -1.87 | -1.06 | 2.27 | -0.01 | 1.49 |
|  | Curacao | Snakebay | SB | 12.1390 | -69.0021 | -0.78 | -6.26 | -3.30 | -3.88 | 4.71 | 0.00 | -0.94 | -2.81 | 7.22 | -2.81 | -1.29 | 5.39 | -1.13 | -1.37 |
|  | Curacao | Vaersenbay | VB | 12.1611 | -69.0112 | -0.79 | -2.29 | -3.08 | -3.51 | 3.48 | 1.12 | -0.96 | -1.87 | 3.47 | -1.87 | -1.29 | 2.64 | -1.13 | -1.22 |
|  | Curacao | Buoy 1 | Z | 12.1259 | -69.0253 | -0.79 | -4.36 | -3.00 | -3.36 | 2.81 | -0.01 | -0.91 | -3.47 | 4.95 | -3.47 | -1.29 | 2.12 | -1.11 | -1.20 |
|  | Dominica | Grande Savane | DM | 15.4369 | -61.4485 | -0.18 | -2.05 | -3.46 | -0.62 | -0.45 | -0.84 | -1.64 | -0.71 | 1.32 | -0.71 | -1.15 | 1.06 | 1.08 | 0.76 |
|  | Tobago | Buccoo Reef | TB | 11.1831 | -60.8334 | -0.13 | -0.42 | -3.56 | 0.66 | 2.50 | -0.77 | -1.39 | 0.62 | 2.99 | 0.62 | -1.24 | 4.12 | -0.41 | -0.54 |
|  | Venezuela | Cayo de Agua | AV | 11.8178 | -66.9306 | -0.57 | 0.89 | -3.68 | -1.15 | 2.88 | 2.20 | -1.18 | -2.11 | 2.99 | -2.11 | -1.29 | 2.93 | -0.86 | -1.14 |
|  | Venezula | Dos Mosquises | BV | 11.7958 | -66.8842 | -0.56 | -0.01 | -3.74 | -1.16 | 2.65 | 1.28 | -1.18 | -1.19 | 2.19 | -1.19 | -1.29 | 4.20 | -0.87 | -1.17 |

**Table S5.** Summary of DISTLM population-scale outputs. Includes the best identified explanation of *Symbiodinium* community variance, and best result for each number of variables (only 1-10 variables shown). AIC = score of model fit (selection criterion), R^2^ = coefficient of determination (% variance explained), RSS= residual sum of squares.

| Model type | AIC score | R^2^ | RSS | No. of variables | Variables included |
| --- | --- | --- | --- | --- | --- |
| Best overall model | 230.83 | 0.54 | 26347 | 7 | dist, ts_acute_0, ts_chron, chla_ave, nit_ave, pho_ave, year |
| *One term model* | 236.61 | 0.19 | 45927 | 1 | ts_chron |
| *Two term model* | 234.89 | 0.28 | 40891 | 2 | ts_acute_0, ts_chron |
| *Three term model* | 233.37 | 0.36 | 36627 | 3 | ts_acute_0, ts_chron, pho_ave |
| *Four term model* | 232.33 | 0.42 | 33305 | 4 | ts_acute_0, ts_chron, chla_ave, pho_ave |
| *Five term model* | 231.59 | 0.46 | 30576 | 5 | dist, ts_acute_0, ts_chron, chla_ave, pho_ave |
| *Six term model* | 231.62 | 0.50 | 28750 | 6 | dist, ts_acute_0, ts_chron, chla_ave, nit_ave, pho_ave |
| *Seven term model* | 230.83 | 0.54 | 26347 | 7 | dist, ts_acute_0, ts_chron, chla_ave, nit_ave, pho_ave, year |
| *Eight term model* | 231.13 | 0.56 | 24988 | 8 | dist,ts_acute_0, ts_chron, chla_ave, nit_ave, pho_ave, depth, year |
| *Nine term model* | 231.61 | 0.58 | 23826 | 9 | dist, ts_acute_0, ts_chron, ts_histor_0, chla_ave, nit_ave, pho_ave, depth, year |
| *Ten term model* | 232.52 | 0.60 | 23028 | 10 | dist, ts_acute_0, ts_chron, ts_histor_0, chla_ave, nit_ave, pho_ave, encl, depth, year |

**Table S6**: Associations between six *Symbiodinium* types hosted by *Orbicella annularis* and environmental factors. These six sub-types all showed significant spatial patterning across the study area. Only significant associations (p<0.05) are listed. *ns* = non-significant.

| *Environmental factor* | *B1* | *B17* | *B1j* | *B10* | *C7a* | *C7* |
| --- | --- | --- | --- | --- | --- | --- |
| Acute thermal stress | *ns* | *ns* | *ns* | *ns* | *ns* | *ns* |
| Acute thermal stress (severe) | *ns* | *ns* | *ns* | *ns* | *ns* | *ns* |
| Chronic thermal stress | *ns* | *ns* | R^2^=0.12, *p=0.049* | *ns* | R^2^=0.22, *p=0.008* | R^2^=0.41, *p=0.0001* |
| History of thermal stress | *ns* | R^2^=0.14,  *p=0.034* | *ns* | *ns* | *ns* | *ns* |
| Turbidity | *ns* | R^2^=0.74,  *p<0.001* | *ns* | *ns* | *ns* | *ns* |
| Salinity | *ns* | *ns* | *ns* | *ns* | *ns* | *ns* |
| Nitrate concentration | *ns* | *ns* | *ns* | *ns* | *ns* | *ns* |
| Phosphate concentration | *ns* | *ns* | R^2^=0.19, *p=0.02* | *ns* | *ns* | *ns* |
| Wave exposure | R^2^=0.21, *p=0.009* | *ns* | *ns* | R^2^=0.15, *p=0.029* | *ns* | *ns* |

**Table S7**: Genotype data for the coral host, *Orbicella annularis*, by site.

| **Location** | **Site Identifier** | **No of samples** | **No. unique genotypes** | **Genotypic diversity** | **% samples with clonemates** | **Identical genotypes** | **Max no. clonemates** | **Clonemate groups hosting identical symbionts (%)** |
| --- | --- | --- | --- | --- | --- | --- | --- | --- |
| Honduras | A | 23 | 21 | 0.91 | 26% | 3 | 2 | 67 |
|  | B | 22 | 20 | 0.91 | 18% | 2 | 2 | 100 |
|  | C | 22 | 12 | 0.55 | 50% | 1 | 11 | 0 |
| Belize | D | 22 | 11 | 0.5 | 68% | 4 | 5 | 100 |
|  | E | 16 | 8 | 0.5 | 75% | 4 | 6 | 50 |
|  | G | 17 | 14 | 0.82 | 35% | 3 | 2 | 33 |
|  | H | 14 | 6 | 0.43 | 64% | 1 | 9 | 0 |
| Bahamas | CI | 16 | 14 | 0.88 | 25% | 2 | 2 | 100 |
|  | EN | 24 | 17 | 0.71 | 38% | 2 | 7 | 0 |
|  | K | 22 | 12 | 0.55 | 64% | 4 | 6 | 75 |
|  | L | 16 | 13 | 0.81 | 31% | 2 | 3 | 100 |
|  | N | 23 | 19 | 0.83 | 30% | 3 | 3 | 67 |
|  | P | 23 | 21 | 0.91 | 22% | 2 | 3 | 0 |
| Nicaragua | NA | 16 | 7 | 0.44 | 75% | 3 | 5 | 67 |
|  | NB | 22 | 11 | 0.5 | 68% | 4 | 6 | 50 |
| Columbia | CM | 11 | n/a | n/a | n/a | n/a | n/a | n/a |
| Cuba | CA | 24 | 11 | 0.46 | 71% | 4 | 9 | 100 |
|  | CB | 23 | 17 | 0.74 | 43% | 4 | 3 | 67 |
|  | CC | 24 | 21 | 0.88 | 25% | 3 | 2 | 33 |
| Cayman | X | 23 | 22 | 0.96 | 9% | 1 | 2 | 0 |
| Dominican Rep. | DR | 5 | 5 | 1 | 0% | 0 | 0 | no clones |
| Jamaica | JA | 18 | 12 | 0.67 | 61% | 5 | 3 | 100 |
|  | JB | 21 | 18 | 0.86 | 29% | 3 | 2 | 100 |
| Barbados | BA | 14 | 12 | 0.86 | 14% | 2 | 2 | 50 |
| BVI | R | 2 | 2 | 1 | 0% | 0 | 0 | no clones |
|  | T | 16 | 13 | 0.81 | 38% | 3 | 2 | 33 |
| Curaçao | SB | 16 | 9 | 0.56 | 69% | 4 | 4 | 50 |
|  | VB | 16 | 8 | 0.5 | 69% | 3 | 5 | 67 |
|  | Z | 18 | 4 | 0.22 | 94% | 3 | 11 | 33 |
| Dominica | DM | 19 | 5 | 0.26 | 95% | 4 | 12 | 50 |
| Tobago | TB | 23 | 9 | 0.39 | 83% | 5 | 7 | 40 |
| Venezuela | AV | 13 | 12 | 0.92 | 31% | 2 | 2 | 100 |
|  | BV | 12 | 11 | 0.92 | 17% | 1 | 2 | 0 |

**References**

[1] Sampayo, E.M., Dove, S. & Lajeunesse, T.C. 2009 Cohesive molecular genetic data delineate species diversity in the dinoflagellate genus *Symbiodinium*. *Molecular Ecology* **18**, 500-519. (doi:10.1111/j.1365-294X.2008.04037.x).

[2] LaJeunesse, T.C. & Trench, R.K. 2000 Biogeography of two species of *Symbiodinium* (Freudenthal) inhabiting the intertidal sea anemone *Anthopleura elegantissima* (Brandt). *Biological Bulletin* **199**, 126-134. (doi:10.2307/1542872).

[3] Thornhill, D.J., LaJeunesse, T.C., Kemp, D.W., Fitt, W.K. & Schmidt, G.W. 2006 Multi-year, seasonal genotypic surveys of coral-algal symbioses reveal prevalent stability or post-bleaching reversion. *Marine Biology* **148**, 711-722. (doi:10.1007/s00227-005-0114-2).

[4] LaJeunesse, T.C. 2002 Diversity and community structure of symbiotic dinoflagellates from Caribbean coral reefs. *Marine Biology* **141**, 387-400. (doi:10.1007/s00227-002-0829-2).

[5] Warner, M.E., LaJeunesse, T.C., Robison, J.D. & Thur, R.M. 2006 The ecological distribution and comparative photobiology of symbiotic dinoflagellates from reef corals in Belize: Potential implications for coral bleaching. *Limnology and Oceanography* **51**, 1887-1897.

[6] Frankin, E.C., Stat, M., Pochon, X., Putnam, H.M. & Gates, R.D. 2012 GeoSymbio: a hybrid, cloud-based web application of global geospatial bioinformatics and ecoinformatics for Symbiodinium-host symbioses. *Molecular Ecology Resources* **12**, 369-373. (doi:10.1111/j.1755-0998.2011.03081.x).

[7] Perry, J.N. 1995 Spatial Analysis by Distance Indices. *Journal of Animal Ecology* **64**, 303-314. (doi:10.2307/5892).

[8] Perry, J.N., Winder, L., Holland, J.M. & Alston, R.D. 1999 Red–blue plots for detecting clusters in count data. *Ecology Letters* **2**, 106-113. (doi:10.1046/j.1461-0248.1999.22057.x).

[9] Foster, N.L. 2007 Population dynamics of the dominant Caribbean reef-building coral, *Montastraea annularis* [Ph.D]. Devon, UK, University of Exeter.

[10] Foster, N.L., Baums, I.B., Sanchez, J.A., Paris, C.B., Chollett, I., Agudelo, C.L., Vermeij, M.J.A. & Mumby, P.J. 2013 Hurricane-driven patterns of clonality in an ecosystem engineer: the Caribbean coral *Montastraea annularis*. *PLoS ONE* **8**, e53283.

[11] Finney, J., Pettay, D., Sampayo, E., Warner, M., Oxenford, H. & LaJeunesse, T. 2010 The relative significance of host–habitat, depth, and geography on the ecology, endemism, and speciation of coral endosymbionts in the genus *Symbiodinium*. *Microbial Ecology* **60**, 250-263.

[12] Iglesias-Prieto, R. & Trench, R.K. 1997 Acclimation and adaptation to irradiance in symbiotic dinoflagellates. II. Response of chlorophyll–protein complexes to different photon-flux densities. *Marine Biology* **130**, 23-33. (doi:10.1007/s002270050221).

[13] LaJeunesse, T.C. 2005 "Species" radiations of symbiotic dinoflagellates in the Atlantic and Indo-Pacific since the miocene-pliocene transition. *Molecular Biology and Evolution* **22**, 570-581.

[14] Garren, M., Walsh, S., Caccone, A. & Knowlton, N. 2006 Patterns of association between *Symbiodinium* and members of the *Montastraea annularis* species complex on spatial scales ranging from within colonies to between geographic regions. *Coral Reefs* **25**, 503-512. (doi:10.1007/s00338-006-0146-1).

[15] Rowan, R., Knowlton, N., Baker, A. & Jara, J. 1997 Landscape ecology of algal symbionts creates variation in episodes of coral bleaching. *Nature* **388**, 265-269.

[16] Thornhill, D.J., Xiang, Y., Fitt, W.K. & Santos, S.R. 2009 Reef endemism, host specificity and temporal stability in populations of symbiotic dinoflagellates from two ecologically dominant Caribbean corals. *PLoS ONE* **4**, e6262.

[17] Kemp, D., Fitt, W. & Schmidt, G. 2008 A microsampling method for genotyping coral symbionts. *Coral Reefs* **27**, 289-293. (doi:10.1007/s00338-007-0333-8).

[18] Santos, S.R., Shearer, T.L., Hannes, A.R. & Coffroth, M.A. 2004 Fine-scale diversity and specificity in the most prevalent lineage of symbiotic dinoflagellates (Symbiodinium, Dinophyceae) of the Caribbean. *Molecular Ecology* **13**, 459-469. (doi:10.1046/j.1365-294X.2003.02058.x).

[19] Green, E.A., Davies, S.W., Matz, M.V. & Medina, M. 2014 Quantifying cryptic *Symbiodinium* diversity within *Orbicella faveolata* and *Orbicella franksi* at the Flower Garden Banks, Gulf of Mexico. *PeerJ* **2**, e386. (doi:10.7717/peerj.386).

[20] Parkinson, J.E., Coffroth, M.A. & LaJeunesse, T.C. 2015 New species of Clade B *Symbiodinium* (Dinophyceae) from the greater Caribbean belong to different functional guilds: *S. aenigmaticum* sp. nov., *S. antillogorgium* sp. nov., *S. endomadracis* sp. nov., and *S. pseudominutum* sp. nov. *Journal of Phycology* **51**, 850-858. (doi:10.1111/jpy.12340).

[21] Andras, J.P., Kirk, N.L. & Drew Harvell, C. 2011 Range-wide population genetic structure of *Symbiodinium* associated with the Caribbean Sea fan coral, *Gorgonia ventalina*. *Molecular Ecology* **20**, 2525-2542.

[22] Edmunds, P., Pochon, X., Levitan, D., Yost, D., Belcaid, M., Putnam, H. & Gates, R. 2014 Long-term changes in *Symbiodinium* communities in *Orbicella annularis* in St. John, US Virgin Islands. *Marine Ecology Progress Series* **506**, 129-144. (doi:10.3354/meps10808).

[23] Thornhill, D., Fitt, W. & Schmidt, G. 2006a Highly stable symbioses among western Atlantic brooding corals. *Coral Reefs* **25**, 515-519. (doi:10.1007/s00338-006-0157-y).

[24] LaJeunesse, T.C., Smith, R.T., Finney, J. & Oxenford, H. 2009 Outbreak and persistence of opportunistic symbiotic dinoflagellates during the 2005 Caribbean mass coral 'bleaching' event. *Proceedings. Biological sciences / The Royal Society* **276**, 4139-4148.

[25] Oxenford, H., Roach, R. & Brathwaite, A. 2008 Large scale coral mortality in Barbados: a delayed response to the 2005 bleaching episode. In *Proceedings of the 11th International Coral Reef Symposium* (Ft. Lauderdale, Florida.

[26] Thornhill, D.J., Lewis, A.M., Wham, D.C. & LaJeunesse, T.C. 2014 Host-specialist lineages dominate the adaptive radiation of reef coral endosymbionts. *Evolution* **68**, 352-367. (doi:10.1111/evo.12270).

[27] Stat, M. & Gates, R.D. 2011 Clade D *Symbiodinium* in scleractinian corals: a nugget of hope, a selfish opportunist, an ominous sign, or all of the above? *Journal of Marine Biology* **2011**. (doi:10.1155/2011/730715).

[28] Pettay, D.T., Wham, D.C., Smith, R.T., Iglesias-Prieto, R. & LaJeunesse, T.C. 2015 Microbial invasion of the Caribbean by an Indo-Pacific coral zooxanthella. **112**, 7513-7518. (doi:10.1073/pnas.1502283112).

[29] Kennedy, E.V., Foster, N.L., Mumby, P.J. & Stevens, J.R. 2015 Widespread prevalence of cryptic *Symbiodinium* D in the key Caribbean reef builder, *Orbicella annularis*. *Coral Reefs* **34**, 519-531. (doi:10.1007/s00338-015-1264-4).

[30] Stat, M. & Gates, R.D. 2011 Clade D *Symbiodinium* in scleractinian corals: a nugget of hope, a selfish opportunist, an ominous sign, or all of the above? *Journal of Marine Biology* **2011**. (doi:10.1155/2011/730715).

[31] Toller, W.W., Rowan, R. & Knowlton, N. 2001a Zooxanthellae of the *Montastraea annularis* species complex: patterns of distribution of four taxa of *Symbiodinium* on different reefs and across depths. *Biological Bulletin* **201**, 348-359.

[32] Toller, W.W., Rowan, R. & Knowlton, N. 2001b Repopulation of zooxanthellae in the Caribbean corals *Montastraea annularis* and *M. faveolata* following experimental and disease-associated bleaching. *Biological Bulletin* **201**, 360-373.

[33] Bouchon, C., Portillo, P., Bouchon-Navaro, Y., Max, L., Hoetjes, P., Brathwaite, A., Roach, R., Oxenford, H.A., O'Farrell, S. & Day, O. 2005 Status of the coral reefs of the Lesser Antilles after the 2005 coral bleaching event. In *Status of Caribbean coral reefs after bleaching and hurricanes in 2005* (eds. C. Wilkinson & D. Souter), GRCMN.

[34] Kemp, D.W., Thornhill, D.J., Rotjan, R.D., Iglesias-Prieto, R., Fitt, W.K. & Schmidt, G.W. 2015 Spatially distinct and regionally endemic *Symbiodinium* assemblages in the threatened Caribbean reef-building coral *Orbicella faveolata*. *Coral Reefs* **34**, 535-547. (doi:10.1007/s00338-015-1277-z).
